# Genomic Architecture may Influence Recurrent Chromosomal Translocation Frequency in the Igh Locus

**DOI:** 10.3389/fimmu.2013.00500

**Published:** 2013-12-30

**Authors:** Amy L. Kenter, Robert Wuerffel, Satyendra Kumar, Fernando Grigera

**Affiliations:** ^1^Department of Microbiology and Immunology, University of Illinois College of Medicine, Chicago, IL, USA

**Keywords:** B cells, Igh locus, chromosomal translocations, AID, genomic structure

## Introduction

B cell lymphomas represent 95% of all lymphomas diagnosed in the Western world and the majority of these arise from germinal center (GC) B cells (1). Recurrent chromosomal translocations involving Ig loci and proto-oncogenes are a hallmark of many types of B cell lymphoma (2). Three types of breakpoints can be identified in Ig loci. Translocation breakpoints adjacent to the D_H_ or J_H_ gene segments form secondary to V(D)J recombination, a process that occurs in early B cell development. Other translocations are located in rearranged V(D)J exons that have acquired mutations indicating that translocation is a byproduct of somatic hypermutation (SHM) which occurs in GC B cells. A third type of translocation is characterized by breakpoints in the *Igh* switch regions, a target for double strand DNA breaks (DSBs) during class switch recombination (CSR) that occurs in mature B cells, both inside and outside the GC. Thus, in B lymphocytes, V(D)J joining, CSR, and SHM create obligate single- or double-strand DNA breaks as intermediates for chromosomal translocations (3, 4).

Activation-induced deaminase (AID) is the enzyme that initiates CSR and SHM (5) by inducing the formation of DSBs in switch (S) regions and mutations in V gene exons (6–10). Studies indicate that non-Ig genes are mistargeted by AID (11, 12) and thereby acquire single and double strand DNA breaks at sites coincident with translocation breakpoints (1, 2). Mature B cells are particularly prone to chromosomal translocations that juxtapose Ig genes and proto-oncogenes, including c-myc [Burkitt’s lymphoma (BL)], Bcl-2 (follicular lymphoma), Bcl-6 (diffuse large cell lymphoma), and FGFR (multiple myeloma) and which are characteristic of human B cell malignancies (2). The mouse plasmacytoma (PCT) T(12;15)(*Igh-myc*) translocation, a direct counterpart of the human BL t(8;14)(q24;q32) translocation, occurs as a dynamic process in mature B cells undergoing CSR and is dependent on the expression of AID (13, 14). Hence, a direct mechanistic link between AID and chromosomal translocations focused to Ig genes has been established.

One of the most puzzling aspects of recurrent chromosomal translocations is that DSBs on two different chromosomes must come into close proximity frequently enough to facilitate the crossover. How do the broken ends located at distal sites in cis or on trans chromosomes come together? Consideration of oncogenic selection, sources of translocation prone DSBs associated with antigen receptor rearrangements in B and T lymphocytes, and the role of DSB persistence in translocations have been recently reviewed [(15, 16) and references therein]. Here we consider the proposition that the spatial organization of mammalian genomes is intrinsically linked to genome stability and modulates the frequency of chromosomal translocations.

## A Model for Recurrent Chromosomal Translocations

Two general models have been proposed to explain the non-random nature of higher order spatial genome organization and the correlation with chromosomal translocations (17). The “contact-first” model posits that translocations require pre-existing physical proximity, whereas, the “breakage-first” model postulates that distant DSBs can be juxtaposed, perhaps through DNA repair machinery. These two theories, the dynamic “breakage-first” and the static “contact-first,” differ fundamentally in their requirement for the presence of DSBs and the mobility of the broken ends.

In the contact-first model only limited local positional motion of DSBs is expected. In the breakage-first model, single DSBs are formed and must undergo large scale movement within nuclei to search for appropriate interaction partners. Although evidence for mobility has been found in yeast systems (18–20), the situation in mammalians cells appears different. In mammalian cells, damaged DNA is largely stationary over time (21–23). However, deprotected telomeres as well as joining of broken DNA ends during V(D)J recombination experience higher mobility (24, 25). Accordingly, the V_H_ subdomain of the *Igh* locus has been described as spatially unstructured (26) although additional studies are required to confirm this conclusion. Nevertheless, the weight of evidence in mammalian systems favors the “contact-first” model in light of the limited spatial mobility of DSBs (27). Comparison of a genomic organization map with sites of chromosomal translocation revealed that the spatial proximity of two DSBs is a dominant factor in determining the translocation landscape genome-wide (28). Therefore, it is useful to examine the disposition of loci within chromatin architecture and how this influences the probability of two DSBs finding each other in nuclear space.

## Three Dimensional Organization of the Mammalian Genome

Emerging evidence indicates that a fundamental property of the mammalian nucleus is the non-random organization of the genome in nuclear space (29). Cytogenetic studies reveal that the mammalian nucleus is occupied by non-randomly positioned genes and chromosomes (30). Together these studies have shown that gene activation or silencing is often associated with repositioning of that locus relative to nuclear compartments and other genomic loci. In this regard, it is relevant that in normal B cells, the breakage sites of several common translocations are more frequently found in close spatial proximity in the nucleus than would be expected based on random positioning (31). A similar relationship between translocation frequency and spatial proximity is observed in BL where the myc locus is on average closest to its most frequent translocation partner, *Igh* (32). The non-random aspect of genome spatial organization in a sub-compartmentalized nuclear space has emerged as a potential contributor to the genesis of chromosomal translocations (23).

The combination of new imaging tools and the comprehensive mapping of long range chromosomal interaction has revealed structural features and biological properties of the three dimensional (3D) genomic organization (33–38). Four features contributing to an ordered 3D organization of eukaryotic genomes have become evident. (1) Individual chromosomes occupy distinct chromosomal territories (CT) with only a limited degree of intermingling (39). (2) The eukaryotic genome is partitioned into functionally distinct euchromatin and heterochromatin (40). (3) Individual genomic loci and elements display preferences for nuclear positioning which correlates well with genomic functions including transcriptional activity and replication timing (39, 41). (4) Distant chromosomal elements associate to form chromatin loops thereby providing a mechanism for long range enhancer function (36, 38, 42). These variables predict that unique and unanticipated spatial genomic relationships may determine unique combinations of chromosomal translocations that may differ in specific tissues and during differentiation.

### Chromosomal looping interactions facilitate CSR

The best studied property of chromatin looping is the spatial proximity of genes and their regulatory elements to establish functional states. Of relevance here is the recognition that chromatin looping influences partner selection during V(D)J recombination (43–45), CSR (46, 47), and may drive specific chromosomal translocation events (28, 48, 49). It is of importance to understand the spatial relationships within the *Igh* locus and how they relate to the preferential expression of Ig gene expression and protect against genome instability. We focus here on CSR because the most prevalent B cell lymphomas arise from GC B cells and are dependent on the expression of AID (1, 13, 14).

**Figure 1:**
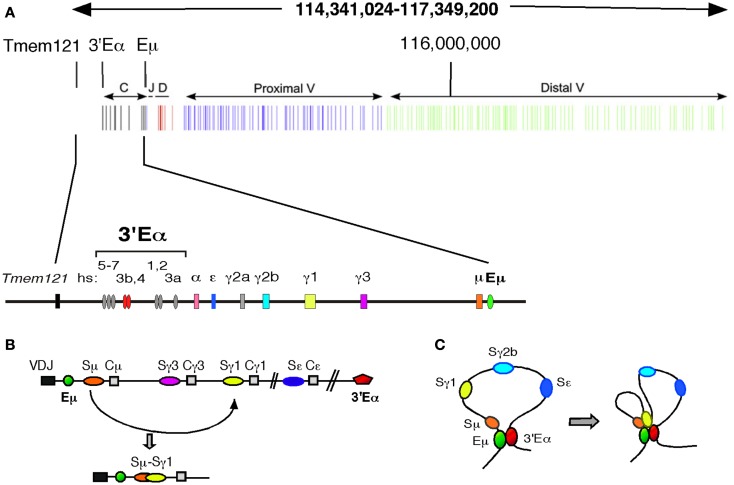
**Long range chromatin looping interactions in the Igh locus facilitate CSR in mature B cells**. **(A)** A schematic map, drawn to scale of the 2.9 Mb *Igh* locus located on chromosome 12 (chr12: 114,341,024–117,349, 200 mm9). The C_H_, J_H_, D_H_, and proximal and distal V_H_ gene segments are indicated. The *Igh* enhancers, 3′Eα and intronic Eμ bracket the C_H_ region gene cluster (top). A schematic showing an expanded segment of the *Igh* locus spanning 220 kb and containing the C_H_ region genes (bottom). The orientation of this map follows the chromosomal organization of the *Igh* locus. **(B,C)** Diagrams of the *Igh* C_H_ locus describing CSR are by convention shown with the Eμ enhancer at the 5′ end. **(B)** CSR promotes diversification of C_H_ effector function while retaining the original V(D)J rearrangement. Within the mouse *Igh* locus, a 220 kb genomic region contains eight C_H_ genes (encoding μ, δ, γ3, γ1, γ2b, γ2a, ε, and α chains) each paired with repetitive switch (S) DNA (with the exception of Cδ). CSR is focused on S regions and involves an intra-chromosomal deletional rearrangement. Germline transcript (GLT) promoters, located upstream of I exon-S-C_H_ regions, focus CSR to specific S regions by differential transcription activation (50, 67). Prior to CSR and upon GLT expression, S regions become accessible to AID attack. AID initiates a series of events culminating in formation of S region specific double strand breaks (DSBs) at the donor Sμ and a downstream acceptor S region (50). DNA DSBs in transcribed S regions are essential for CSR. Here, Sμ and Sγ1 acquire AID induced DSBs and engage in CSR to form recombinant Sμ/Sγ1 regions. **(C)** In mature B cells Eμ:3′Eα interactions create a long range chromatin loop encompassing the C_H_ domain of the *Igh* locus (left). Upon B cell activation with LPS + IL4, long range chromatin interactions directed by the GLT promoters and *Igh* enhancers creates spatial proximity between Sμ and the downstream Sγ1 region locus (46). This spatial proximity facilitates recombination between the broken S regions and creates a matrix of chromatin contacts, which stabilize the locus during the recombination transaction.

Class switch recombination promotes diversification of C_H_ effector function while retaining the original rearranged V(D)J exons. The mouse *Igh* locus spans 2.9 Mb within which a centromeric 220 kb genomic region contains eight C_H_ genes (encoding μ, δ, γ3, γ1, γ2b, γ2a, ε, and α chains) each paired with repetitive S DNA (with the exception of Cδ) (Figure 1A). CSR is focused on S regions and involves an intra-chromosomal deletional rearrangement (Figure 1B). Germline transcript (GLT) promoters, located upstream of I exon-S-C_H_ regions, focus CSR to specific S regions by differential transcription activation (9, 50). The I-S-C_H_ region genes are embedded between the Eμ intronic and 3′Eα enhancers (51). Chromosome conformation capture (3C) studies reveal that in mature resting B cells the transcriptional enhancer elements, Eμ and 3′Eα, engage in long range chromatin looping interactions (46, 47) (Figure 1C). B cell activation leads to induced recruitment of the GLT promoters to the Eμ:3′Eα complex that in turn facilitates GLT expression and supports S/S synapsis (46).

The 3′Eα regulatory region plays a significant role in mediating the spatial structure of the *Igh* locus during CSR as well as promoting genome stability (52). Targeted deletion of hs3b,4 within 3′Eα abolishes GLT expression and GLT promoter:3′Eα and Eμ:3′Eα looping interactions (46, 53, 54). AID initiates a series of events ending in creation of S region specific DNA DSBs at the donor Sμ and a downstream acceptor S region to create S/S junctions and facilitate CSR (7). S regions targeted by AID for DSB formation are transcriptionally active. Chromatin looping across this region ensures proximity between two S regions targeted for DSB creation and recombination (Figure 1C). Thus, CSR is dependent on 3D chromatin architecture mediated by long range intra-chromosomal interactions between distantly located transcriptional elements that serves to tether broken chromosomal DNA together during the CSR reaction.

Chromosome conformation capture (3C, 4C, 5C, and Hi-C) based studies indicate that the most probable chromatin interactions are the most proximal ones and the probability of contact decreases with distance. Correspondingly, alignment of genomic organization maps with sites of chromosomal translocation generated in Hi-C and 4C studies have shown that translocations are enriched in cis along single chromosomes containing the target DSB and in trans in a manner related to pre-existing spatial proximity (28, 55). The positional immobilization of DSBs in the *Igh* locus, for example, should render the probability of successful translocation as the product of the frequency of each DSB at the sites of crossover and the frequency with which these sites are synapsed in physical space (28). In B lymphocytes *c-myc/Igh* translocations occur in trans and may represent a failure of stringent spatial sequestration of AID induced DSBs to within the *Igh* locus (56, 57).

### Dynamic chromatin interactions and the genesis of chromosomal translocations

Chromosomal translocation frequency as reported by genome-wide translocation sequencing is determined by the frequency of AID induced DSB at translocation targets, factors that contribute to synapsis of broken loci, and circumvention of DNA repair functions that facilitate intra-chromosomal DSB joining (55–58). Are recurrent chromosomal translocations simply the result of a stochastic process related to the probability of contact between AID induced DSBs? Tagging single loci with Lac operon (LacO) arrays, as well as photobleaching and photoactivation experiments, have shown that interphase chromatin is locally mobile but rarely moves over long distances (59–61). However, lamina associated domains are large genomic regions that are in intermittent molecular contact with the nuclear lamina indicating a dynamic spatial architecture of chromosomes (62). Chromatin looping, clustering, and compartmentalization are dynamic and responsive to developmental and environmental cues. Functionally dynamic chromatin responses include formation of transcription and replication factories, and nuclear relocation of loci during development (63–66). The looping interactions spanning the *Igh* locus during CSR and in the presence of DSBs may also be dynamic and to some degree transient. In a dynamic chromosomal setting, DSBs present in an *Igh* locus that lacks Eμ:3′Eα tethering, for example, would be at high risk of re-joining to sites outside the *Igh* locus along chromosome 12 and at lower frequency to sites on other chromosomes. The dynamism of chromosomal transactions are not yet fully described and represent the next forefront for investigation to appreciate constraints and variables of genome stability and instability.

## Author Contributions

Drs. Robert Wuerffel, Satyendra Kumar, Fernando Grigera, and Amy L. Kenter were all involved in developing the ideas regarding long range chromatin interactions and dynamics that are the subject here and all have critiqued and agree to the contents of this piece. Amy L. Kenter wrote the article.
